# Supplementary material for: Short- and Long-Term Effects of Suboptimal Selenium Intake and Developmental Lead Exposure on Behavior and Hippocampal Glutamate Receptors in a Rat Model
Source: Nutrients. 2022 Aug 10;14(16):3269. doi: 10.3390/nu14163269 (PMC9416673; doi:10.3390/nu14163269)
Supplement: Supplementary file 1 [file nutrients-14-03269-s001.zip › Supplementary Figure Legends.pdf]

Supplementary Figure 1. Somatic growth and sensorimotor development in neonatal rat pups. (A) Body weight and (B) body length; (C) righting reflex; (D) latency to reach the nest arm and (E) percentage of time spent in nest arm at PND 11 in the Homing test. Data are sex-pooled represented, except for righting reflex; (A,B) * $p < 0.05$ Se Subopt/Veh vs Se Subopt/Pb; (C) $ $p < 0.05$ Se Subopt/Pb females vs Se Subopt/Pb males; (D) $p < 0.05$ Se Subopt vs Se Opt.

Supplementary Figure 2. Effect of Se diet on the expression and post-synaptic trafficking of glutamatergic receptors. Hippocampal expression of GluN2B (A) subunit of NMDA receptor in HOMO and GluA2 subunit of AMPA receptor in homogenate (HOMO) (B) and TIF (C) of PND 70 rats fed with fed with optimal or suboptimal Se diet. Data are represented as ratio on actin levels ($n \geq 3$). One way ANOVA, * $p < 0.05$, *** $p < 0.001$, **** $p < 0.0001$.

Supplementary Figure 3. Sex influence in the expression of glutamatergic receptors in juvenile rats fed with optimal Se diet and exposed to Pb. Expression of NMDA and AMPA receptors and PSD-95 in homogenate of PND 23 female (A, B, C) and male (D, E, F) rats fed with optimal selenium diet and exposed to ~1.25 mg/kg body weight Pb per day. Data were normalized on actin levels and are represented as % of vehicle ($n \geq 6$ for glutamatergic receptors, $n \geq 4$ for PSD-95); Unpaired t-test, not significant.

Supplementary figure 4. Effect of suboptimal Se diet ad Pb exposure on glutamatergic receptors expression and post-synaptic trafficking in female juvenile rats. Expression of glutamatergic NMDA and AMPA receptors and PSD-95 in the homogenate (A, B, C) and TIF (D, E) of PND 23 hippocampi of female rats fed with suboptimal Se diet. Data were normalized on actin levels and are represented as % of vehicle ($n \geq 4$); Unpaired t-test, not significant.
