# Supplementary material for: Short- and Long-Term Effects of Suboptimal Selenium Intake and Developmental Lead Exposure on Behavior and Hippocampal Glutamate Receptors in a Rat Model
Source: Nutrients. 2022 Aug 10;14(16):3269. doi: 10.3390/nu14163269 (PMC9416673; doi:10.3390/nu14163269)
Supplement: Supplementary file 1 [file nutrients-14-03269-s001.zip › Supplementary Material.pdf]

Supplementary Material

Short- and long-term effects of suboptimal selenium intake and developmental lead exposure on behavior and hippocampal glutamate receptors in a rat model

Tartaglione AM[1a], Serafini MM[2a], Ferraris F[3], Raggi A[3], Mirabello A[1], Di Benedetto R[3], Ricceri L[1], Midali M[2], Cubadda F[3], Minghetti L[4], Viviani B[2]*, Calamandrei G[1].

[1] Centre for Behavioral Sciences and Mental Health, Istituto Superiore di Sanità (ISS), Rome, Italy

[2] Department of Pharmacological and Biomolecular Sciences, Università degli Studi di Milano, Milan, Italy

[3] Department of Food Safety, Nutrition and Veterinary Public Health, Istituto Superiore di Sanità (ISS), Rome, Italy

[4] Research Coordination and Support Service, Istituto Superiore di Sanità (ISS), Rome, Italy

[a] These authors have contributed equally to this work

[*] Corresponding author

1 Material and Methods

Plasma and milk Se determinations

Ultrapure grade water obtained from a Milli-Q Element purification system with 0.22 μm filters was used in the analytical determinations (Millipore, Molscheim, France). The other reagents used were 67-69% v/v ultrapure grade nitric acid (HNO_3) (Carlo Erba Reagenti, Milan, Italy) and ultrapure 30% v/v grade hydrogen peroxide (H_2O_2) (Sigma Aldrich).

The Selenium (Se) calibration solution and Rhodium (Rh) internal standard solution were obtained by dilution from certified standard solutions (High Purity Standards, Charleston, SC, USA 1000 ± 3 μg/ml).

An 8800 ICP-MS Triple Quad spectrometer from Agilent Technologies (Tokyo, Japan) was used to determine total Se. The same sample introduction system described above was used. To eliminate potential spectral interferences on the selenium signal, oxygen was used as a reaction gas in mass shift and the analytical masses were SeO^+ at mass 94 and 96; the operating conditions are shown in **Table S1**.

Plasma samples were diluted 1:50 with a 0.5% v/v HNO_3 solution. Milk samples were digested in a microwave system (UltraWAVE Single Reaction Chamber Microwave Digestion System, Milestone, Bergamo, Italy) and diluted 1:5 before analytical determinations. Total Se quantitative analysis was carried out with external calibration; the calibration range was 0.1-25 μg/L. Rh was used at a

concentration of 0.5 µg/L. An ultrapure water sample of the same volume as the samples, collected in the same Eppendorf containers, was used as the procedural blank. The procedural blank (n = 3) was subtracted in the calculation of the sample concentrations.

As for blood Pb, the analytical determinations were carried out in a clean room environment and, to assess measurement accuracy, analytical quality control procedures were adopted. Seronorm Serum L1 was used for as reference material for Se plasma and it was subjected to the same analytical procedure as the samples till the final instrumental determination. The measured value was 64.6 µg/L (s.d. 1.2 µg/L, $n = 3$), in good agreement with the certified value of 59.2 µg/L (acceptance range: 53.6-64.8 µg/L). NIST 1549 (Non-Fat Milk Powder) and BCR 063R (Skin Milk) were used as certified reference materials for Se milk and were subjected to the same analytical procedure as the samples till the final instrumental determination. The measured value for NIST 1549 was 110 µg/L (s.d. 1 µg/L, $n = 6$), compared to a certified value of 110 µg/L (acceptance range: 100-120 µg/L), whereas for BCR 063R the measured value was 131 µg/L (s.d. 2 µg/L, $n = 3$), compared to an indicative value of 125 µg/L.

Blood Pb determinations

Ultrapure grade water obtained from a Milli-Q Element purification system with 0.22 µm filters was used in the analytical determinations (Millipore, Molscheim, France). The other reagents used were 67-69% v/v ultrapure grade nitric acid (HNO_3) (Carlo Erba Reagenti, Milan, Italy) and Triton X-100 (J.T. BAKER).

The Lead (Pb) calibration solutions and the Rhodium (Rh) internal standard solution were obtained by dilution from certified standard solutions (High Purity Standards, Charleston, SC, USA 1000 ± 3 µg/ml).

An 8800 ICP-MS Triple Quad from Agilent Technologies (Tokyo, Japan) was used to determine total Pb. The instrument is equipped with a reaction/collision cell (ORS3 cell) between two quadrupole analyzers (Q1 and Q2). The sample introduction system consisted of a concentric PFA nebulizer, a double-pass (Scott type) PFA nebulization chamber cooled to 2 °C and a platinum injector. The analytical mass used was ^{208}Pb; the operating conditions of the instrument are shown in **Table S2**.

Blood samples were diluted 1:50 using a solution consisting of 0.2% v/v HNO_3 and 0.005% v/v Triton X-100. The quantitative analysis of the total Pb was carried out with the standard additions method. The calibration range with the standard addition method was 0.1-5 µg/L. Rh (internal standard) was used at a concentration of 0.5 µg/L.

An ultrapure water sample of the same volume as the samples, collected in the same Eppendorf containers, was used as the procedural blank. The procedural blank ($n = 3$) was subtracted in the calculation of the sample concentrations.

Seronorm Whole Blood L1 was used as reference material to verify the accuracy of the analytical determinations. This material was subjected to the same analytical procedure as the samples till the final instrumental determination. The measured value was 15.0 µg/L (s.d 0.5 µg/L, $n = 3$), in excellent agreement with the certified value of 14.8 µg/L (acceptance range 13.8-15.8 µg/L).

Morris Water Maze in young adults (MWM, PND 68-72)

MWM was performed in a circular black pool (diameter 210 cm) filled with 24 °C water rendered opaque by the addition of atoxic acrylic paint (Giotto, Italy). An escape platform (diameter 10 cm) was submerged 2 cm below the water level. Animals used distal visual-spatial cues (posters on the walls of the room) to find the hidden escape platform located in the center of the target quadrant. Each rat underwent two phases of MWM: a spatial learning phase (training) of four days duration followed by a single Probe trial 24 h after the last training trial. Training_During the spatial learning phase (four days with four consecutive trials per day) rats were released into the pool from one of the four starting points (one per each quadrant) and trained to find the hidden escape platform. The order of the sequence was changed pseudo-randomly between days. A trial was finished when the animal found the escape platform or when 60 s had elapsed. If a rat failed to find the platform, it was gently guided to the platform by the experimenter. Rats remained on the platform for 30 s. After each trial rats were dried and when the session finished they were returned to their colony room.
Probe Trial_24 hours after training, spatial memory was assessed with a probe trial in which the escape platform was removed and rats were released from the quadrant opposite to target quadrant (which contained the escape platform during the training phase) and allowed to swim for 60 s searching for it. Latency to reach the escape platform, time spent in each quadrant, latency and distance moved to first entry to target quadrant and path efficiency were recorded and analyzed by ANY-maze software (Stoelting Europe, Dublin, Ireland).

2 Supplementary Figures and Tables

Table S1. Operating conditions used for Se determination by ICP-MS/MS.

INSTRUMENTAL PARAMETERS	OPERATING CONDITIONS
**Power RF**	1550 W
**Nebulizer**	Esi PFA-LC
**Spray Chamber**	Scott PFA inert kit
**Nebulizer gas flow**	0.82 L min-1
**Makeup gas**	0.31 L min-1
**Nebulizer pump**	0.10 rps
**Acquisition mode**	MS/MS reaction
**Reaction gas**	Oxygen
**Percentage of the reaction gas**	20%
**Sampling time**	30 sec

**Time / mass integration**	2 sec
**Masses selected from Q1**	78, 80, 103
**Masses selected from Q2**	94, 96, 103

Table S2. Operating conditions used for Pb determination by ICP-MS / MS.

INSTRUMENTAL PARAMETERS	OPERATING CONDITIONS
**Power RF**	1550 W
**Carrier gas flow**	0.95 L min^{-1}
**Q1 bias**	-2 V
**Octopole bias**	-6 V
**Acquisition mode**	Full Quant MS/MS
**Nebulizer pump**	0.10 rps
**Sampling time**	30 sec
**Time / mass integration**	2 sec
**Masses selected from Q1**	208, 103
**Masses selected from Q2**	208, 103

Table S3. Reproductive performances.

Group		Number of pups	Sex ratio	Body weight at birth
Se Subopt	Veh	15.286 ± 1.976	0.872 ± 0.460	6.491 ± 0.553
	Pb	13.750 ± 2.915	0.997 ± 0.791	6.608 ± 0.998
Se Opt	Veh	15.300 ± 2.669	1.161 ± 0.619	6.392 ± 1.151

Pb	11.444 ± 3.005	0.968 ± 0.518	6.390 ± 0.518

Table S4. Effect of Se diet on the expression and post-synaptic trafficking of glutamatergic receptors in adult rats: Optimal *versus* Suboptimal Se at PND 70.

	Receptor	Subunit	Optimal Se (Mean ± SEM)	n Optimal Se	Suboptimal Se (Mean ± SEM)	n Suboptimal Se	p-value
**HOMO**	**NMDA**	*GluN2A*	0.33 ± 0.02	11	0.51 ± 0.09	11	0.0817
		GluN2B			*see supplementary figure 2A*		
		GluN1	0.24 ± 0.03	12	0.35 ± 0.08	11	0.1229
	**AMPA**	*GluA1*	0.22 ± 0.02	11	0.37 ± 0.10	11	0.2926
		GluA2			*see supplementary figure 2B*		
**TIF**	**NMDA**	*GluN2A*	0.31 ± 0.04	12	0.50 ± 0.09	11	**0.0374 ***
		GluN2B	0.21 ± 0.03	13	0.32 ± 0.07	12	0.1183
		GluN1	0.65 ± 0.09	13	0.81 ± 0.11	10	0.2444
	**AMPA**	*GluA1*	0.16 ± 0.05	11	0.48 ± 0.09	12	**0.0008 *****
		GluA2			*see supplementary figure 2C*		

Table S5. Effect of Se diet on the expression and post-synaptic localization of PSD-95, selenoprotein and Se transporter in adult rats: Optimal *versus* Suboptimal Se at PND 70.

**Protein**	**Optimal Se** (Mean ± SEM)	**n Optimal Se**	**Suboptimal Se** (Mean ± SEM)	**n Suboptimal Se**	**p-value**
PSD-95 HOMO	0.49 ± 0.07	12	0.85 ± 0.13	11	**0.0216 ***
SEPP1 HOMO	0.28 ± 0.06	12	0.44 ± 0.04	12	0.5823
LRP8 HOMO	0.45 ± 0.04	12	0.60 ± 0.10	11	0.1660
LRP8 TIF	0.41 ± 0.06	10	0.60 ± 0.04	11	0.3632

Table S6. Effect of Optimal Se diet and Pb exposure on glutamatergic receptors expression and post-synaptic trafficking in adult rats: Optimal Se + Pb at PND 70.

HOMO	Receptor	Subunit	Vehicle (Mean ± SEM)	n Vehicle	Pb (Mean ± SEM)	n Pb	p-value
**FEMALE**	**NMDA**	*GluN2A*	100 ± 15.8	5	82.4 ± 11.0	5	0.3887
		GluN2B	100 ± 22.7	5	86.4 ± 7.5	4	0.6232
		GluN1	100 ± 14.3	4	88.3 ± 2.9	4	0.4547
	**AMPA**	*GluA1*	100 ± 10.7	4	109.9 ± 15.1	4	0.6123
		GluA2	100 ± 25.6	4	74.7 ± 11.4	4	0.4015
**MALE**	**NMDA**	*GluN2A*	100 ± 4.8	7	110.9 ± 8.6	6	0.2732
		GluN2B	100 ± 8.5	7	89.2 ± 2.7	7	0.2820
		GluN1	100 ± 4.2	6	120.7 ± 8.5	7	0.0626
	**AMPA**	*GluA1*	100 ± 8.3	7	124.8 ± 11.5	6	0.1196
		GluA2	100 ± 8.9	8	109.8 ± 8.9	7	0.4516
**TIF**	**Receptor**	**Subunit**	**Vehicle** (Mean ± SEM)	**n Vehicle**	**Pb** (Mean ± SEM)	**n Pb**	**p-value**
**FEMALE**	**NMDA**	*GluN2A*	100 ± 10.4	5	126.6 ± 12.2	4	0.1396
		GluN2B	100 ± 15.3	5	120.9 ± 15.5	5	0.3659
		GluN1	100 ± 18.4	4	138.2 ± 7.7	5	0.0764
	**AMPA**	*GluA1*	100 ± 16.3	5	125.3 ± 2.1	4	0.2156
		GluA2	100 ± 15.6	4	136.7 ± 8.9	4	0.0877
**MALE**	**NMDA**	*GluN2A*	100 ± 15.0	7	78.0 ± 18.1	6	0.3655

	GluN2B	100 ± 19.9	7	62.2 ± 12.4	6	0.1503
	GluN1	100 ± 9.7	7	110.5 ± 4.8	4	0.4575
**AMPA**	*GluA1*	100 ± 15.9	7	168.1 ± 61.7	7	0.3063
	GluA2	100 ± 23.0	8	96.5 ± 30.7	7	0.9270

Table S7. Effect of Optimal Se diet and Pb exposure on the expression of PSD-95 in adult rats: Optimal Se + Pb at PND 70.

	**Protein**	**Vehicle** (Mean ± SEM)	**n Vehicle**	**Pb** (Mean ± SEM)	**n Pb**	**p-value**
**FEMALE**	*PSD-95*	100 ± 20.9	4	157.3 ± 17.1	5	0.0692
**MALE**	*PSD-95*	100 ± 14.9	7	104.5 ± 13.0	6	0.9967

Table S8. Effect of Suboptimal Se diet and Pb exposure on glutamatergic receptors expression and post-synaptic trafficking in adult rats: Suboptimal Se + Pb at PND 70.

**HOMO**	**Receptor**	**Subunit**	**Vehicle** (Mean ± SEM)	**n Vehicle**	**Pb** (Mean ± SEM)	**n Pb**	**p-value**
**FEMALE**		*GluN2A*	100 ± 13.5	6	106.8 ± 22.4	6	0.8001
	**NMDA**	*GluN2B*	100 ± 34.1	5	141.1 ± 36.0	6	0.4349
		GluN1	100 ± 15.5	6	86.7 ± 8.5	5	0.4986
	**AMPA**	*GluA1*	100 ± 21.1	5	94.4 ± 7.7	5	0.8104
		GluA2	100 ± 26.4	5	113.6 ± 18.0	5	0.6820
**MALE**	**NMDA**	*GluN2A*	100 ± 18.2	5	86.8 ± 18.4	5	0.6230
		GluN2B	100 ± 16.5	5	109.0 ± 20.6	6	0.7490

		GluN1	100 ± 36.0	5	100.9 ± 37.4	6	0.9975
	**AMPA**	*GluA1*	100 ± 19.4	5	57.5 ± 22.1	5	0.1874
		GluA2	100 ± 21.4	5	103.1 ± 33.4	6	0.9430

**TIF**	**Receptor**	**Subunit**	**Vehicle** (Mean ± SEM)	**n Vehicle**	**Pb** (Mean ± SEM)	**n Pb**	**p-value**
**FEMALE**	**NMDA**	*GluN2A*	100 ± 30.1	6	126.2 ± 38.7	6	0.6040
		GluN2B	100 ± 42.2	4	285.3 ± 121.2	6	0.2669
		GluN1	100 ± 33.7	5	89.9 ± 46.6	5	0.8653
	**AMPA**	*GluA1*	100 ± 24.4	5	101.3 ± 32.7	5	0.9749
		GluA2	100 ± 41.0	5	175.5 ± 39.8	6	0.2220
**MALE**	**NMDA**	*GluN2A*	100 ± 27.0	6	53.0 ± 13.1	5	0.1779
		GluN2B	100 ± 23.2	5	46.1 ± 9.2	5	0.0631
		GluN1	100 ± 18.3	5	83.4 ± 17.1	5	0.5267
	**AMPA**	*GluA1*	100 ± 14.6	5	49.5 ± 12.1	6	**0.0247 ***
		GluA2	100 ± 12.0	6	63.4 ± 5.4	5	**0.0291 ***

Table S9. Effect of Suboptimal Se diet and Pb exposure on the expression of PSD-95 in adult rats: Suboptimal Se + Pb at PND 70.

	**Protein**	**Vehicle** (Mean ± SEM)	**n Vehicle**	**Pb** (Mean ± SEM)	**n Pb**	**p-value**
**FEMALE**	*PSD-95*	100 ± 20.1	5	248.9 ± 75.8	6	0.1162
**MALE**	*PSD-95*	100 ± 44.7	6	106.5 ± 43.5	6	0.8137

3 Supplementary Results

Spatial learning and memory in Morris Water Maze

Training_A main effect of training days [$F(3,216) = 119.420$ $p<0.001$] indicated that all rats significantly decreased their escape latencies to reach the hidden platform across days.

Probe trial (24 hr memory) ANOVA, with quadrant as within-subject factor and diet, treatment and sex as between-subject factors, was performed to analyze the amount of time that experimental groups spent in each one of the quadrant of the MWM when platform was removed. Results showed that the main effect of quadrant [$F(3,216) = 29.710$ $p<0.001$]. All rats spent significantly more time in the quadrant where the platform was located (target quadrant) than in the remaining quadrants. No effects of Se diets, Pb or their interaction wasfound on spatial learning and memory in this task.
