# Supplementary figures and images for: Short- and Long-Term Effects of Suboptimal Selenium Intake and Developmental Lead Exposure on Behavior and Hippocampal Glutamate Receptors in a Rat Model
Source: Nutrients. 2022 Aug 10;14(16):3269. doi: 10.3390/nu14163269 (PMC9416673; doi:10.3390/nu14163269)

**A**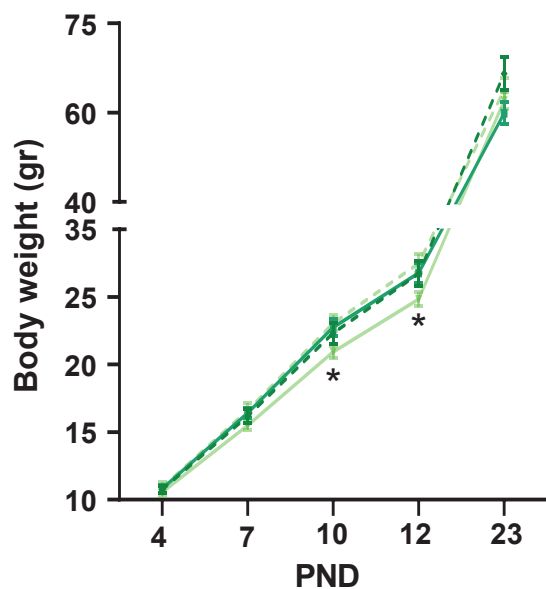**B**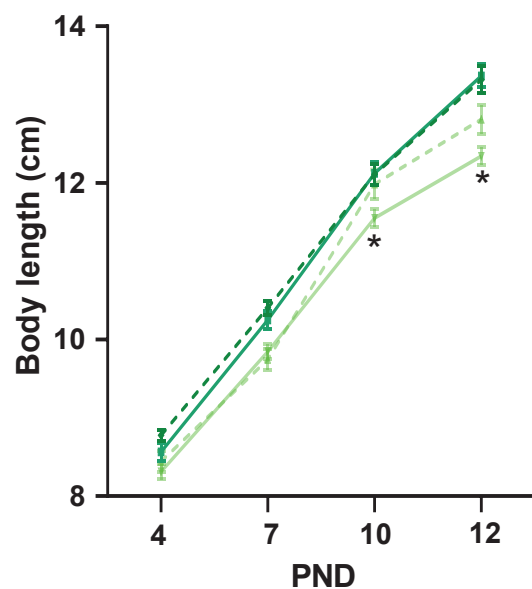

Se Opt/Veh Se Opt/Pb Se Subopt/Veh Se Subopt/Pb

**C**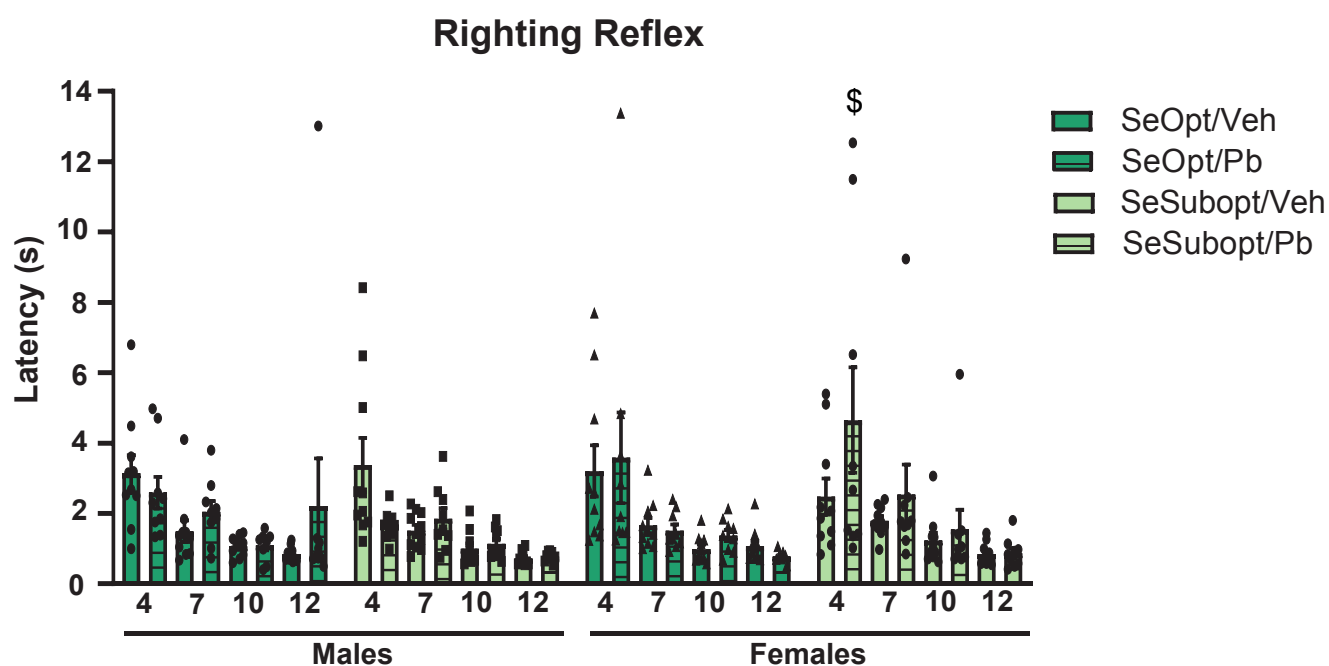**D**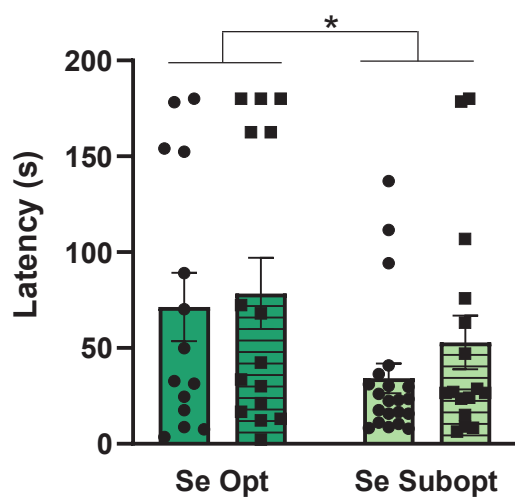**E**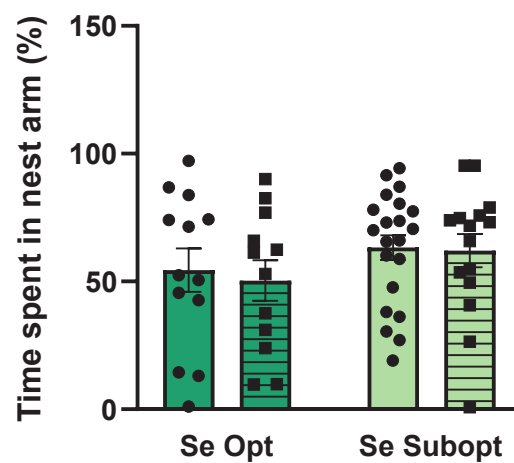

Supplement: Supplementary file 1 [file nutrients-14-03269-s001.zip › Supplementary Figure 1.pdf]

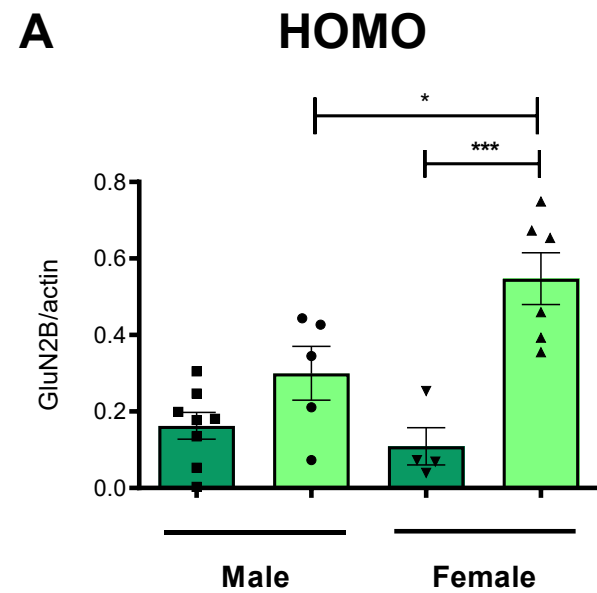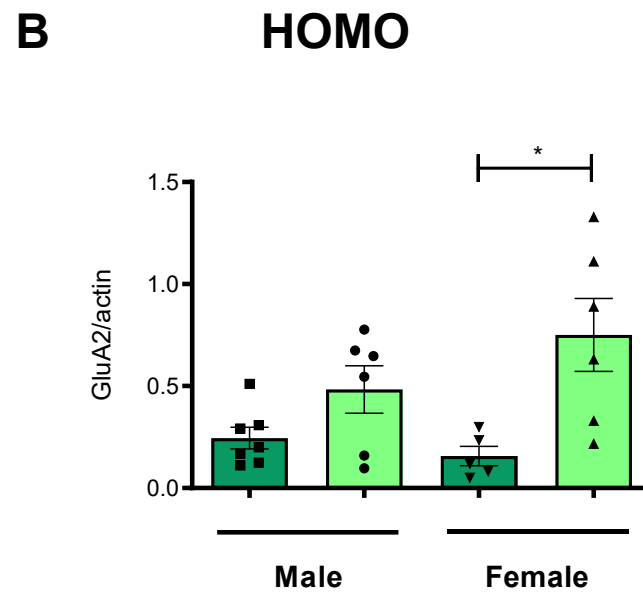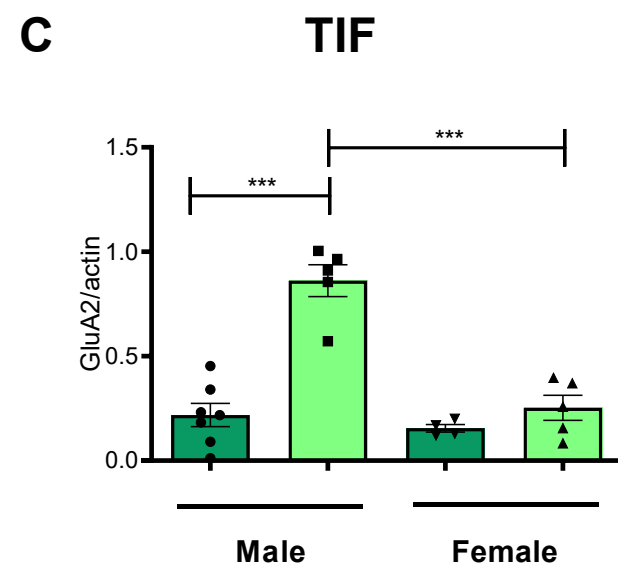

Se Opt/Veh  
Se Subopt/Veh

Supplement: Supplementary file 1 [file nutrients-14-03269-s001.zip › Supplementary Figure 2.pdf]

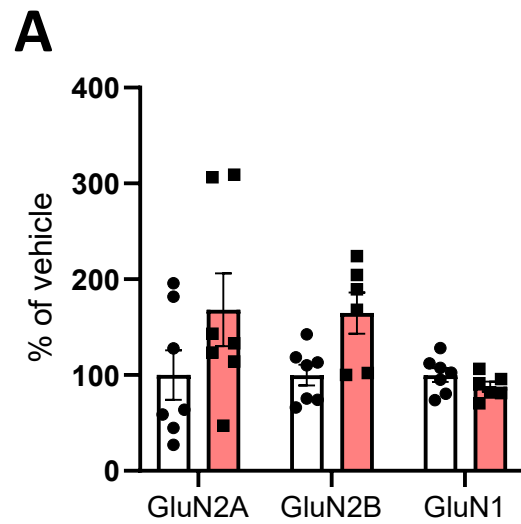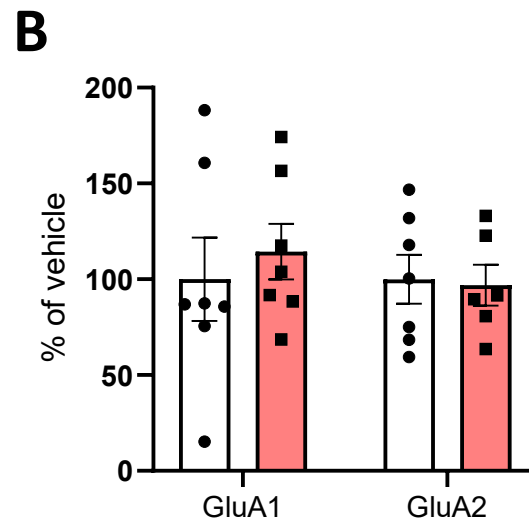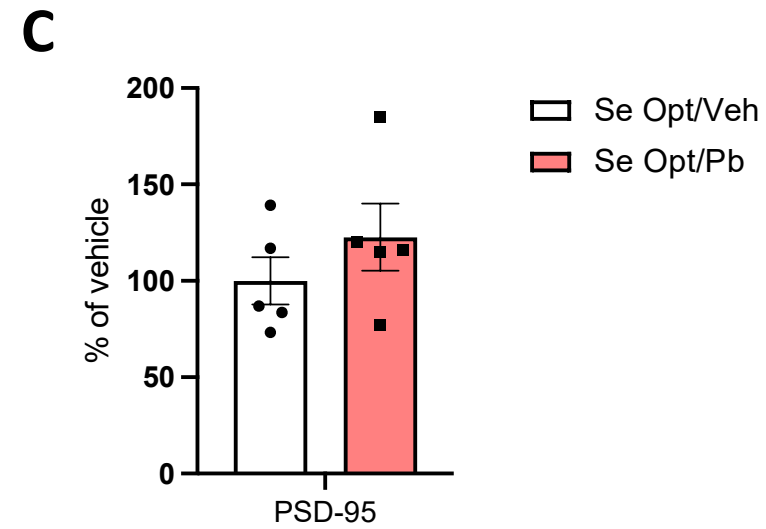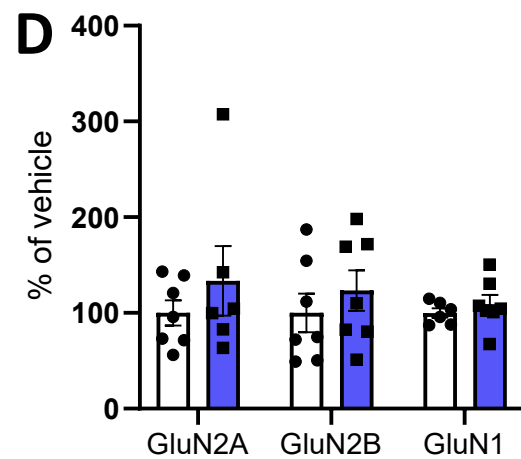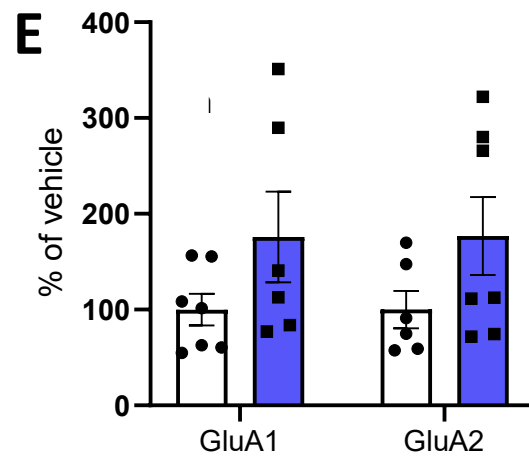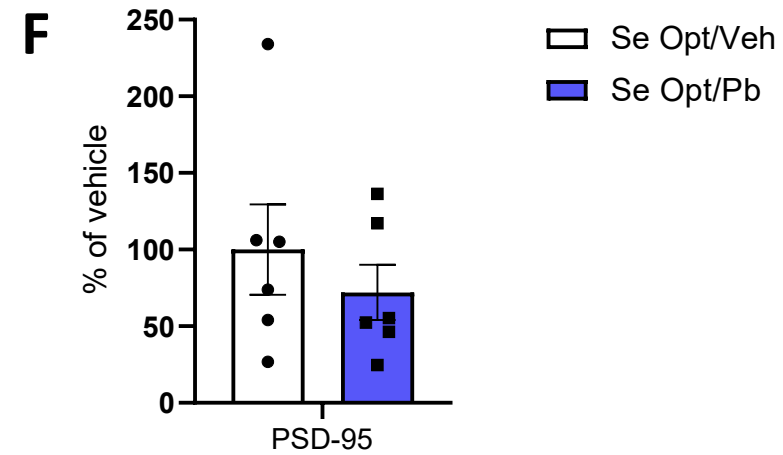

Supplement: Supplementary file 1 [file nutrients-14-03269-s001.zip › Supplementary Figure 3.pdf]

**A**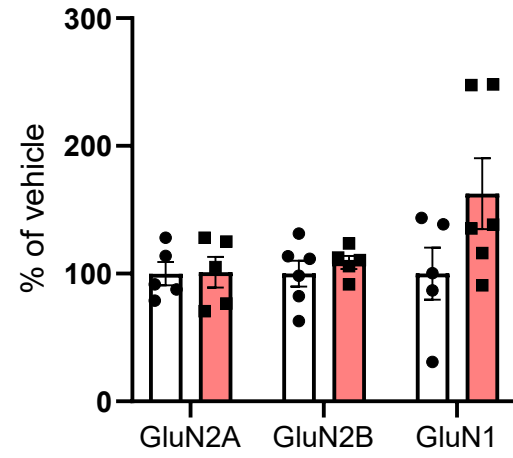**B**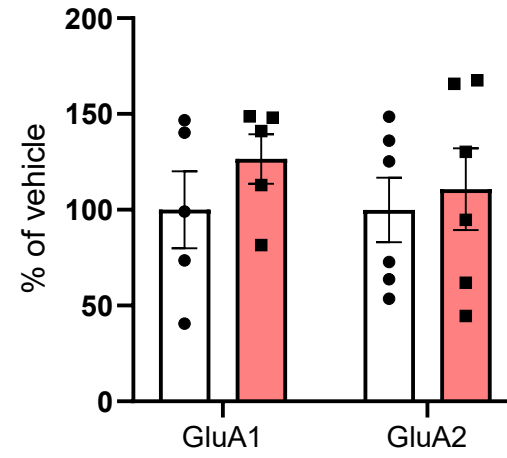**C**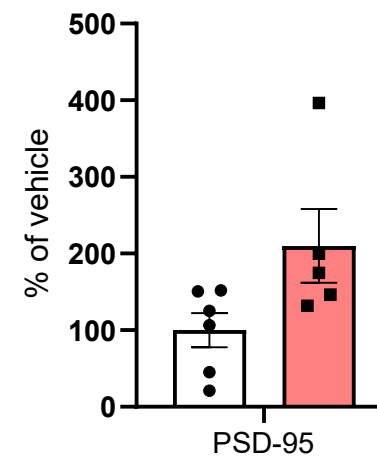**D**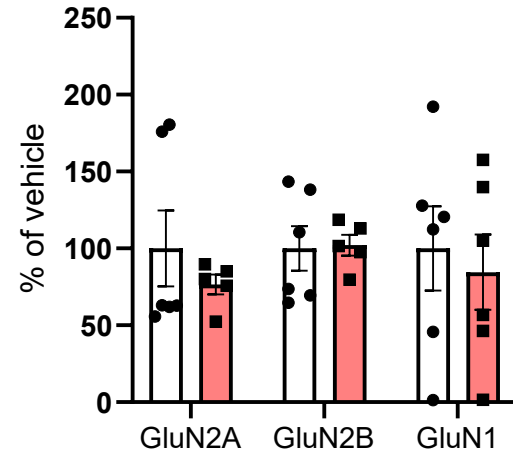**E**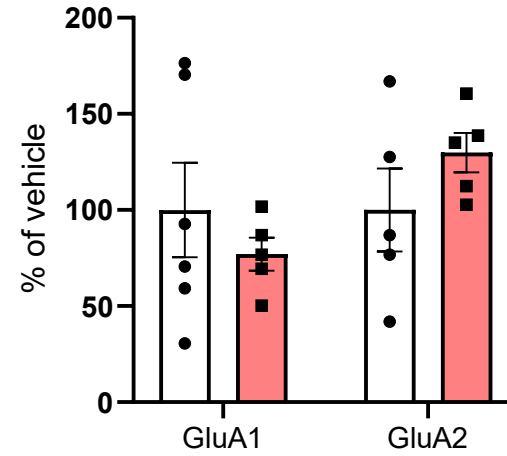

□ Se Subopt/Veh  
■ Se Subopt/Pb

Supplement: Supplementary file 1 [file nutrients-14-03269-s001.zip › Supplementary Figure 4.pdf]
